# Dialysis, Distress, and Difficult Conversations: Living with a Kidney Transplant

**DOI:** 10.3390/healthcare10071177

**Published:** 2022-06-23

**Authors:** Clare McKeaveney, Helen Noble, Aisling E. Courtney, Sian Griffin, Paul Gill, William Johnston, Alexander P. Maxwell, Francesca Teasdale, Joanne Reid

**Affiliations:** 1 School of Nursing and Midwifery, Queen’s University Belfast, Belfast BT9 7BL, UK; c.mckeaveney@qub.ac.uk (C.M.); helen.noble@qub.ac.uk (H.N.); 2 Renal Unit, Belfast Health & Social Care Trust, Belfast BT9 7ER, UK; aisling.courtney@belfasttrust.hscni.net; 3 Department of Nephrology and Transplantation, Cardiff & Vale University Health Board, Cardiff CF14 4XW, UK; sian.griffin2@wales.nhs.uk; 4 School of Healthcare Sciences, Cardiff University, Cardiff CF24 0AB, UK; gillp3@cardiff.ac.uk; 5 Northern Ireland Kidney Patients Association, Belfast BT9 7AB, UK; willjohnston1@hotmail.co.uk; 6 Centre for Public Health, Queen’s University Belfast, Belfast BT12 6BA, UK; a.p.maxwell@qub.ac.uk; 7 Kidney Care, Alton GU34 1EF, UK; francescacgt2013@gmail.com

**Keywords:** qualitative, kidney, transplant, holistic, wellbeing, interpretative phenomenological analysis

## Abstract

*Background*: Providing holistic care to kidney patients is important; however, without full consideration of the perspectives of people living with a kidney transplant, the provision of truly ‘holistic healthcare’ cannot be possible. It is imperative to understand patient experiences by including kidney patients in key strategies and future renal service planning. Ignoring these important patient views means that there is a significant risk of inappropriate renal service provision and lack of adequate support, impacting overall health. The aim of this study was to develop an in-depth understanding of the lived experiences of kidney transplant recipients. *Methods*: A total of 23 participants were recruited between two regional nephrology units within the United Kingdom via clinical gatekeepers. In-depth interviews were undertaken. Interviews were digitally recorded, transcribed verbatim, and subjected to interpretative phenomenological analysis. *Results*: Two themes emerged: “managing ongoing fears of dialysis, distress, and COVID-19” and “dealing with difficult conversations”. *Conclusions*: Renal healthcare professionals need to understand more than the biological impact of receiving a kidney transplant. Understanding the holistic and multidomain experiences that these participants experience will help healthcare professionals to recognize the needs of this group and ensure more responsive psychosocial care.

## 1. Introduction

Kidney transplantation is the preferred treatment for eligible patients with end-stage kidney disease (ESKD) although dialysis remains the predominant therapy across the globe [1]. The World Health Organization estimated that approximately 97,000 kidney transplants were performed worldwide in 2018. However, considerable variation exists in access to and use of kidney transplantation, with the highest rates in European and Nordic countries [2]. Many patients aim to restore a sense of “normality” to their lives with a functioning kidney transplant [3]. When successful, kidney transplantation reduces mortality and improves quality of life [4]. Kidney transplantation is also associated with improved physical functioning, greater engagement in social and recreational activities, higher independence, and enhanced ability to work when compared with patients receiving other forms of renal replacement therapy such as dialysis [5].

However, kidney transplantation can be associated with an overestimation of physical, psychological, and social quality of life, leading to increased levels of distress [3]. Individuals living with a transplant continue to face significant unmet physical and psychological challenges involving a new way of life [6]. The impact of indefinite therapy with immunosuppressive medications and associated side-effects, adherence to infection prevention, and ongoing kidney function monitoring and routine clinic appointments [7] can cause significant patient burden. Equally, individuals are at an increased risk of developing psychopathological and psychosocial problems post transplantation as concerns, for example, about graft failure are often forever in the background of most recipients [8,9]. These latter issues are receiving greater scientific and medical attention because of their association with poorer adherence to post-transplant pharmacological treatment and a higher probability of graft rejection [10].

After kidney transplantation, optimal self-management requires individuals to take responsibility to manage their symptoms, treatment, and psychosocial and lifestyle changes. However, poor self-management is common in kidney transplantation with relatively high rates of nonadherence to medications, diet, and exercise [11]. Qualitative research has played an important role in generating important insights into the multidimensional nature of self-management requirements highlighting the need for tailored holistic interventions [12]. However, there are very few effective self-management interventions in kidney transplantation which aim to integrate treatment and life goals, with most focusing on medication adherence [7,11]. Therefore, a need to develop an in-depth understanding of the experiences of those living with a kidney transplant continues to inform appropriate self-management strategies comprehensively and holistically [9].

To date, no studies have used an IPA. This method is particularly useful for understanding under-researched phenomena or perspectives. Unlike other methodologies, it is a meticulously idiographic and hermeneutic phenomenological approach that provides a detailed examination of personal lived experiences to make sense of a given phenomenon [13]. Using this methodology, the holistic experiences of living with a kidney transplant across renal units in the United Kingdom were explored.

## 2. Materials and Methods

Design: This study used a qualitative methodology underpinned by Heideggerian phenomenology, which focuses on interpreting and understanding the meaning of lived experiences to gain an in-depth exploration of participant experiences [14,15]. Interpretative phenomenological analysis (IPA) was selected as the most suitable method of qualitative enquiry that can facilitate researchers to construct insightful interpretative accounts of experiences, which are “complex, ambiguous, and emotionally laden” (p. 41, [16]). This methodology ensures a depth of inductive, interpretive analysis, which is grounded firmly in a close examination of what the participant has said [16,17].

Setting: The study was conducted at two regional nephrology units within the United Kingdom.

Study participants: A purposive sample of 23 adults who received a kidney transplant in last 5 years were recruited across two regional sites within the United Kingdom. Individuals were eligible if they had their first kidney transplant in adulthood and were between 6 months and 5 years post-transplant regardless of transplant type (e.g., living donor, deceased, pre-emptive) or previous dialysis modality (e.g., peritoneal dialysis (PD), hemodialysis (HD)). Clinical gatekeepers identified and completed informed consent with participants prior to interview.

Study materials: Semi-structured interview guides were developed in collaboration with the research team (see Supplementary File). Interviews were conducted online or over the telephone with similar data collection techniques in phenomenological research supported within the literature [18] and as per Health Research Authority COVID-19 guidelines [19]. Interviews used an inductive format and utilized nondirective, open-ended questions that can facilitate starting and maintaining a conversation. A semi-structured interview guide was used flexibly within each interview, the appropriateness of which is outlined in the literature [20]. Interviews continued until data saturation was achieved [21]. All interviews were digitally recorded and transcribed verbatim [22]. Interviews were conducted between the start of June 2020 and October 2020. Interview time ranged from 39 min to 128 min (average time 55 min).

Analysis: Data were analyzed according to the principles of IPA [16]. Transcripts were read and analyzed by searching for points of descriptive, linguistic, and conceptual note, in relation to the research aim. Emergent themes were clustered into tables and compared across participants. Whilst analysis was rigorous and sensitive to the context of these individual participants [23], it should be noted that interpretations here are bound by the experiences and sense making of this particular sample. The qualitative nature of these findings adds to our understanding of what it is like to live with a kidney transplant. Analysis was undertaken by the first author (C.M.) and audited by additional authors (H.N. and J.R.). Any disagreements about interpretations were discussed until an agreement was reached.

## 3. Results

Of the 23 interviews, the average age of participants was 49.4, most participants had an experience of dialysis (HD *n* = 7; PD *n* = 7; HD & PD *n* = 1; no dialysis *n* = 8), and 12 participants received a living kidney transplant (of which eight were pre-emptive); the remaining 11 participants received a deceased kidney transplant.

Qualitative analysis elucidated the impact of living with a kidney transplant. On the basis of these findings, the collective experiences are presented across two key themes, which are “managing ongoing fears of dialysis, distress, and COVID-19” and “dealing with difficult conversations”. This section presents each theme using illustrative quotations from the participants’ transcripts.

**Theme 1: Managing ongoing fears of dialysis, distress and COVID-19**

Interviews helped to demonstrate that transplantation is not an explicable event via which a participant’s life is unequivocally re-established or transformed. Participants expressed being extremely grateful and part of something extraordinary, and they related their experience of transplantation to “nothing short of a medical marvel”. However, transplantation was also “[an] unbelievable trauma…” prolonging an existential crisis whereby neither the physical toll nor the psychological burden of kidney failure was eliminated by receipt of a transplant. Regardless of how long a participant was living with kidney disease, the immediacy for transplantation left participants feeling powerless, fearful, and uncertain about their future. The transplant journey was described as a “living numbness”, as well as a sense of shock and being overwhelmed at times, with feelings of instability. Of note, not all participants started dialysis; however, most participants described immense challenges preparing themselves psychologically and physically for the possibility of dialysis, transplantation, and dying. Albeit life-sustaining, dialysis was perceived to be something to be avoided at all costs and associated with a rapid decline in participant functionality and wellbeing,

*“… [sitting in the waiting room] so [I’m] scared… I mean I’m sat next to a guy here who’s got no leg. He’s in a wheelchair because of it. He’s on a dialysis machine. He has a kidney and it failed. I’ve got a guy over the other side, the same happened to him. He’s been on dialysis for five, six years. He has to come to hospital three times a week. I mean I am looking around and thinking [scared]… you know what I mean.”*

Post-transplant participants reflected on a lack of personalized participant information and felt less informed about their disease on matters of severity, as well as future self-management. At initial diagnosis, few participants recalled being told that their condition would eventually require dialysis or kidney transplantation. Participants also described varying and surprising surgical outcomes after transplantation. Removal of the dialysis catheter represented an important step; however, previous fistulas left unforgiving “lumps and bumps” (e.g., aneurysm formations). Surgical scars were expected; however, being able to feel the placement of the new kidney was unexpected. Immunosuppressant medication side-effects were also difficult to manage, and adherence required technological assistance,

*“… my hair started to fall out and, as a woman, this really upsets me. I asked to stop [that] medication but I was told [my hair would] eventually stop [falling out] but [my hair] will never be the same again… to get me into the mind set of taking those tablets, [I] had to use my phone to remind me… I rarely forget now because my phone tells me morning and night…”*

Fears regarding when, not if, the transplant would fail never escaped a participant. Participants also reported a real and unrelenting fear of returning to or having to start “invasive treatment” in the form of dialysis, at some point in their future. Over time, these worries became less prominent; however, they were easily brought to the forefront by symptoms such as pain. Those transplanted just before or during the COVID-19 pandemic also described a combined sense of wellness and frustration as shielding was advised for all participants. It was important to protect their kidney transplant by adhering to strict shielding, cleaning procedures, and isolation. Many described not seeing their children and partners, which sometimes involved living away from the family home, leading to increased feelings of anxiety, depression, and paranoia,

*“It didn’t help the fact that, when I was literally due to go back to work, we got put in lockdown… Because then I became overly paranoid about leaving the house. I can’t leave the house. I can’t hurt the kidney. I don’t want to risk it. I don’t want to risk this virus. I don’t want to risk it. So going back to work last week, I’ve never suffered with panic attacks before or anxiety. And I was a blubbery mess just walking into the doors. Or even sat in the car park. I struggled really badly walking back into work last week.”*

**Theme 2: Dealing with difficult conversations**

Communicating to others about their disease was extremely difficult. Participants recounted numerous reasons why they tended to “kept a lot from [loved ones]”, as they “didn’t want to worry anyone” and that others would “… never really fully understand, completely, really”. Participants also described immense pressure from healthcare professionals to ask family and friends to become living donors. Sometimes, a participant did not have anyone to ask. In general, conversations about living donation were less likely to be initiated by the participant; conversations could take several years to bring up with loved ones and sometimes only occurred by chance. Conversely, when conversations took place resulting in a negative experience (non-match outcomes or decline to offer a kidney), participants were unclear how to manage ongoing or sometimes broken relationships,

*“I think, prior to the [transplant] operation… the [doctor] asked you to ask people if they wanted to give you their kidney. Wow! I couldn’t ask anybody in my life. I couldn’t ask [anybody]. And I never did.”*

Participants reflected on the communicative pressures experienced between participants and those with a living donor. The receipt of a kidney from a deceased donor was considered to sometimes carry a greater sense of relief for participants, whereby potential emotional and physical burdens that would be placed on loved ones would be removed. Post-transplant, participants described a lack of readily available resources for their respective living donors; this included addressing challenges around their physical recovery, as well as their living donors’ long-term psychological recovery,

*“… Because my wife, for example, has now got one kidney. The information that she got was nonexistent. And she fell through a gap, I’m sure. However, there’s no literature, standard literature [location-specific] that said, you have donated. You’ve got one kidney now. You really do need to look after it. Because if it starts failing you haven’t got another one to take over capacity. The GP raised an issue about my wife’s kidney function, and she had to explain to him that she had donated her kidney to me… things like that weren’t helpful to the process.”*

Family members were regarded as the main source of emotional and physical support; however, over time, relationships with partners had changed, and friends had become less available. Participants depicted a sense of loneliness post-transplant that had slowly crept in. Starting new relationships also seemed hampered for participants. Younger transplant recipients described avoiding new relationships due to a fear of disclosing their illness. Participants described experiencing rejection and believed ill health had the potential to or had already interfered with personal and romantic relationships,

*“… I sort of made this conscious decision to not go out with anybody. Because I thought it’s too much to take on for anybody to come in. I just was like very… I’ll deal with relationships and all that after my transplant.”*

Participants described managing a wide range of socioeconomic challenges alone. This included reducing or exiting work or taking earlier than expected retirement as a result of needing treatment for ESKD. Often, this created worries about financial instability, loss of routine, and changes to one’s identity. Younger transplant recipients (<40 years old) described a lack of age-specific information, and they were more likely to do “their own research” on issues relating to their disease and its impact on their physical and social life. They reported current transplant information and, in some cases, staff “didn’t really connect to [their] personal [situation]”. Issues included sensitive and complex topics such as pregnancy and sexual intimacy, which were not addressed by clinicians, nor did participants feel comfortable to approach staff,

*“We weren’t eligible for adoption because at any point this kidney could fail and I would be sick again… in the midst of it all, I never thought it was appropriate, with everything the doctors were doing for me, you know keeping me alive, to start talking about getting pregnant.”*

Not having the “right [kind] of information” from clinicians impacted participants’ ability to effectively support and manage their transplant. Information sessions organized by the transplant team for participants to share experiences and ask questions were described as helpful. However, there was pressure not to get “too personal” or ask “the scary questions”. This feeling carried over into individual sessions with transplant coordinators where participants continued to feel overwhelmed with which questions to ask and to whom. The importance of balancing the provision of transplant information to avoid distress was noted by participants; however, some felt there was not enough information on the variability of post-transplant outcomes preventing development and creation of future self-goals,

*“A big thing for me was, before the [transplant] operation, we were told that conceiving a child would be a struggle and difficult… as for life after transplant, I didn’t know what I wanted because I didn’t know what to expect.”*

Participants reported they would continue to rely heavily on their renal transplant team so much that they were unlikely to report to other medical professionals. For example, when referred for psychological support, several participants found themselves receiving support outside renal care. Many felt conversations around transplant recovery with nonrenal specialists lacked the unique understanding and knowledge of a kidney transplant journey to fully support their psychological needs. Participants strongly advocated for “in-house” psychosocial care,

*“I do think that there does need to be some kind of support network or support thing that needs to be there for the mental aspect of it. I think they are great about the diet side, they are great about the medication side, and all of that. It’s just the mental health of it. Just because you’ve had the transplant doesn’t mean that everything has been fixed, and sometimes you can unearth things that you didn’t even know were there sometimes, as well. It’s not being ungrateful to say that you are feeling down after your transplant… it’s just saying, I am still getting over what has happened to me.”*

## 4. Discussion

The results of this qualitative study expand on existing literature regarding psychosocial distress experienced by kidney transplant recipients [24,25,26,27] by providing a more in-depth understanding of particular concerns experienced. Participants perceived graft failure as life-threatening, causing varying degrees of anxiety of dialysis initiation, re-dialysis, re-transplantation, or even immediate death. Ruminating thoughts about death and dying amplified a living numbness, which was only further exacerbated by a new susceptibility to the COVID-19 infection. Equally, participants reflected on the psychosocial challenges before and after their transplantation, which included difficulties around treatment decision-making, avoiding conversations about living donation, reducing work, rethinking relationships, and a failure to identify future life goals adding to an already isolating existence. These experiences helped to demonstrate that living with a kidney transplant cannot be separated into a discrete time (i.e., post-transplant) and requires an understanding of the totality of living with a kidney transplant (i.e., life pre-transplant).

Kidney transplantation is associated with extreme physiological and psychological stressors [28]. Managing patients’ distress has been recognized as integral to clinical outcomes, as well as healthcare resource use [29]. Distress can impact recovery and rehabilitation, resulting in negative health outcomes post-transplant [30], including an increased risk of developing post-traumatic stress disorder. Recent research has documented elevated rates of PTSD in kidney transplant recipients [31] with previous dialysis duration and a level of perceived suffering from dialysis as important contributors post-transplant [28]. Of note, patients undergoing dialysis tend to experience the lowest quality-of-life outcomes post-transplant, and, as most patients with ESKD dialyze before transplant, a greater acknowledgement of the impact of this experience is required [32]. Unlike other transplanted populations, kidney patients are also more likely to experience improvements in depression and anxiety with a pre-transplant psychological intervention, suggesting unique psychological needs in this patient population [30].

The COVID-19 pandemic further demonstrates the urgent need to provide psychological support to this patient population. Participants described increased anxiety and depressive symptoms during the COVID-19 pandemic. This work helps to highlight the mental health impact of the pandemic on individuals managing extreme risks of infection and prolonged isolation. Research on COVID-19 and clinically extremely vulnerable patient populations continues to grow, with studies reporting an increased prevalence of phobic anxieties, depressive reactions, and sleep disorders [33]. National renal guidelines have mandated timely patient-centered psychosocial care [29], emphasizing the importance of appropriate staff training to embed necessary support for patients, via both formal and informal support pathways [34]. For example, renal clinicians trained in motivational interviewing, a technique used to foster patient engagement and empowerment, has shown beneficial effects in ESKD and could be a useful tool in identifying and addressing emotional distress via informal pathways [35,36,37,38]. Despite recommendations for early screening, immediate and specialist access to more formal psychological support continues to require substantial investment in building a renal psychosocial workforce [39].

Difficult conversations about renal disease and transplant were an interesting finding in this study, demonstrating that there continues to be a lack of good evidence to inform renal support services on how best to meet the holistic needs of kidney transplant recipients [9]. Most distressing are conversations around living donation, whereby participants were set the task of identifying and discussing living donation with loved ones despite receiving limited information and support. Few qualitative studies have explored the impact of “direct recruitment” of a living donor. Much research has focused on recipient–donor health and relationship outcomes [40]. However, reluctance to approach or recruit donors by participants is a barrier to transplantation [26,27]. It is important that renal healthcare professionals have a deeper understanding of the patients’ psychological, emotional, and social status to help develop a tailored support plan for coping with conversations about living donation. In addition, renal healthcare professionals are pivotal in the provision of education and counselling for individuals with ESKD and living with a kidney transplant; however, they require adequate knowledge, training, and time to conduct this appropriately in the patient-centered manner that is required [35,36,37,38].

This study also found an absence of age-sensitive information relating to relationships, family planning, work, finances, and social life. Younger participants felt they were unable to approach renal clinicians about a wide range of topics impacting their kidney transplant journey. It is important to be able to identify and manage any patient modifiable factors relating to mental health and quality of life. These issues can directly impact patient self-management, increasing the risk of medication nonadherence and graft failure. As younger transplant recipients are a high-risk group for transplant failure [41,42,43], there continues to be a need to better understand barriers to kidney health for this small but vulnerable patient population. Prüfe and colleagues highlighted that “as patients’ need for support is not limited to medical questions… the roles of psychologists and social workers in a multidisciplinary setting need to be discussed and strengthened” (p. 9, [44]). Such multidisciplinary work has been previously recommended [39]. Alternative strategies have included digital intervention which has shown some promising improvements in self-management [45], however, adolescent, and young adult kidney transplantation groups require further investigation [46,47].

Of note, this study included a heterogeneous sample of participants despite a focus on a select group (e.g., number of years post-transplant). Therefore, results are not transferable to the entire population of participants living with a kidney transplant. These findings help to reinforce the importance of understanding the lived experiences and the need for holistic support for patients living with a kidney transplant [48]. Aligning this within a standardized multidisciplinary renal workforce, there is a need to develop novel tools to enhance patient education and psychosocial wellbeing, providing a wide range of options accounting for life-stage, future goals, support needs, and experiences throughout the treatment journey [7]. Future work should also seek to develop these through codesigned partnerships with patients and healthcare professionals and using mixed-method approaches to test acceptability and effectiveness.

## 5. Conclusions

This study investigated the lived experience of adult participants living with a kidney transplant. The “participant journey” was the central theme, recommending the need for better integration of the totality of the participant experience into participant information and psychological services in renal healthcare.

## Data Availability

The data presented in this study are available on reasonable request from the corresponding author. The data are not publicly available as it contains information that could compromise the privacy of research participants.

## Supplementary Materials

The following supporting information can be downloaded at https://www.mdpi.com/article/10.3390/healthcare10071177/s1: Table S1: Semi-structured interview schedule example.
