# Supplementary material for: Dialysis, Distress, and Difficult Conversations: Living with a Kidney Transplant
Source: Healthcare (Basel). 2022 Jun 23;10(7):1177. doi: 10.3390/healthcare10071177 (PMC9321787; doi:10.3390/healthcare10071177)
Supplement: Supplementary file 1 [file healthcare-10-01177-s001.zip › healthcare-1737228-supplementary.pdf]

Table S1. Semi-structured interview schedule example.

Topic	Example questions
Open invitation	Tell me a little about your treatment journey?
Emotional	Can you tell me what feelings you experience day to day living with a kidney transplant?
Social	Can you describe to me how you feel your social life has changed since your transplant?
Physical	What has been the impact on your body?
Financial	Can you tell me about the financial practicalities you experience living with a kidney transplant?
Support	Can you describe to me what coping skills you have used?
COVID-19	Can you describe any specific concerns at the beginning of the coronavirus outbreak?<br>Have your concerns changed since the beginning of the coronavirus outbreak?
